# Highly effective and reusable nanometal/carbon-coated snail shell for the sequestration of metronidazole: decontamination and disinfection

**DOI:** 10.1007/s11356-025-36801-w

**Published:** 2025-08-29

**Authors:** James Friday Amaku, Fanyana M. Mtunzi

**Affiliations:** Vaal University of Technology, Vanderbijlpark, 1911 Gauteng South Africa

**Keywords:** Metronidazole, *Achatina fulica*, Thermodynamics, Regeneration, Antimicrobial, Nanocomposite

## Abstract

This study investigates the adsorption efficiency of metronidazole (MTZ) from aqueous solutions using snail shell (SSB) and carbon-coated zinc oxide-modified snail shell (SCZ) nanocomposites. Key factors influencing adsorption capacity, including pH, contact time, adsorbent dose, MTZ concentration, and temperature, were systematically evaluated. The maximum adsorption capacities were 25.96 mg g^−1^ for SSB and 35.13 mg g^−1^ for SCZ, with optimal adsorption occurring at pH 3 after 180 min of contact. Kinetic analysis revealed that the pseudo-second-order model best fit the experimental data, while adsorption isotherms followed the Langmuir model for SSB and the Freundlich model for SCZ, indicating different adsorption behaviors. Thermodynamic analysis showed that adsorption was spontaneous, endothermic, and entropy-driven. MTZ desorption was effectively achieved using ethanol, demonstrating good reusability of the adsorbents. This study highlights the potential of SCZ nanocomposites as an effective, sustainable solution for removing MTZ from aqueous systems.

**Supplementary Information:**

The online version contains supplementary material available at 10.1007/s11356-025-36801-w.

## Introduction

Stakeholders responsible for healthy, habitable, and sustainable aquatic ecosystems have demonstrated a great deal of interest in investigating the increasing introduction of emerging contaminants (ECs) into the water bodies (Morin-Crini et al. 2022). Owing to the persistence and limited knowledge of their potential toxicological implication, ECs such as hormones, medicines, endocrine-disrupting chemicals, and personal care products, among others, are a potential threat to the sustainability of potable water (Saidulu et al. 2021).

Considering the significant risks of pharmacological compounds to human health and biodiversity (de Jesus et al. 2015). An intensive effort has been channeled to the identification of pharmaceutical compounds in aquatic matrices (Jakimska et al. 2014). The application of antibiotics in human or veterinary medicine drives the vast production of this class of drugs, and this could be associated with their persistence in surface water (Caneschi et al. 2023). As a famous medication, metronidazole is a nitroimidazole that is commonly used as an anti-infective medication for the treatment of illnesses brought on by anaerobic bacteria and parasites (Chua 2017). Prior to discharge, it is essential to eliminate metronidazole from industrial effluent, as this will reduce the bioaccumulation of non-biodegradable metronidazole and its metabolites in the aquatic environments (Patel et al. 2021). Due to the devastating implications of metronidazole on human health and aquatic organisms, metronidazole was carefully chosen as the pharmaceutical model for this study.

Wastewater treatment techniques such as nanofiltration (Egea-Corbacho et al. 2019; Moreira et al. 2022), adsorption (Gil et al. 2019; Krishnan et al. 2017, 2015, Rathi and Kumar 2021), coagulation (Matamoros and Salvadó 2013), and advanced oxidation ultraviolet light (Yu et al. 2019) have been explored for the reduction or elimination of hazardous micro-pollutants. Among the itemized techniques, adsorption is regarded to be the most affordable and effective technique for wastewater treatment (Cheng et al. 2021). The technique is also considered user-friendly and void of secondary pollutants. On the other hand, the effectiveness of adsorption is dependent on the quality of the adsorbent employed (Pourhakkak et al. 2021). Studies on the fabrication of activated carbon via the carbonization of agricultural wastes and its application for the elimination of EC via the adsorption process revealed the effectiveness and the limitations (high cost of production and regeneration difficulty) of activated carbon as an adsorbent in the wastewater remediation process (Ukanwa et al. 2019; Wu et al. 2020). Numerous affordable and readily sourced adsorbent materials have been studied for the elimination of pharmaceuticals from industrial effluents, including activated carbon (Ahmadfazeli et al. 2021), biochar (Cheng et al. 2021), ash (Abbas and Abbas 2021), chitosan (Asgari et al. 2020), MWCNTs (Kariim et al. 2020), MOFs (Kalhorizadeh et al. 2022), silica (Szentmihályi et al. 2022), composite (Nasseh et al. 2019), rice husk, TiO_2_ (Neghi et al. 2019), clay (Kalhori et al. 2017), nanoparticles (El Bouraie and Ibrahim 2021), metal oxides (El-Sayed et al. 2014), zeolite (Al-Musawi et al. 2022), graphene oxide (Manjunath et al. 2017), and nanohydroxyapatite (Sarfi et al. 2024),

The application of biochar in a variety of electronics, including engineering materials and environmental remediation processes, has garnered more attention recently (Allohverdi et al. 2021). Biochars are carbonaceous materials that are produced using organic-based resources via the pyrolytic approach (Schmidt and Wilson 2012). The application of this material for water purification purposes has been reported by many authors (Gupta et al. 2022). Surface modification of biochar is essential for its effectiveness in trapping contaminants from wastewater (Kamali et al. 2021). Chemical treatment of biochar or the surface coating of the biochar using nanometals has demonstrated synergism (Wang and Wang 2019). Additionally, zinc oxide nanoparticles (ZnONPs) are known to possess superior characteristics for application in different fields (Siddiqi et al. 2018). It has also shown the capacity to function as an adsorbent for the elimination of different types of contaminants from wastewater (Primo et al. 2020).

*Achatina fulica* Snail is classified into class, domain, family, genus, and kingdom as *Gastropoda, Eukaryota, Achatinidae, Lissachatina,* and *Animalia*, respectively (Thiengo et al. 2010). Snails are mostly eaten as food in some West African countries, and shells are discarded as waste. Locally, snail shells are employed as a building material due to their mechanical strength (Muneer et al. 2018). It is composed of an organic matrix and calcium carbonate. Hence, it can function as a support in adsorbent fabrication.

This study presents, for the first time, the fabrication and application of ZnO nanoparticle-decorated, carbon-coated *Achatina fulica* (snail) shells as a low-cost, sustainable, and efficient adsorbent for the removal of metronidazole from aqueous solutions. By utilizing an abundant biowaste material enhanced with nanotechnology, the research provides a novel adsorbent design, explores its adsorption performance under various physicochemical conditions, and offers new insights into its adsorption mechanisms through comprehensive isotherm, kinetic, and thermodynamic studies.

## Materials and methods

Metronidazole (C_6_H_9_N_3_O_3_, 99.97%), %), zinc acetate dihydrate (Zn(CH_3_CO_2_)_2_·2H_2_O, 98%), and other analytical grade reagents employed in this study were obtained from Sigma-Aldrich and used without further treatment.

### Preparation of adsorbent

Snailshells were sourced from Ahia-eke market located at Ibeku, Umuahia, Abia State, Nigeria. After the snailshells underwent a series of steps, including washing, air-drying for 7 days, pulverizing, and sieving through a 120 µm mesh screen, they were placed in an airtight plastic bag and kept for future use. About 15 g of the processed snailshells was added to a slurry of brown sugar (3 g, 20 cm^3^ of H_2_O), and the mixture was oven-dried for 24 h. In a Carbolite Gero tubular furnace, the snailshell/sugar mixture was heated at a rate of 10 °C/min and pyrolyzed for one hour at 350 °C at a rate of 30 cm_3_ min^−1^ of N_2_. Thereafter, the carbon-coated snailshells (CSS) were kept in storage for future use. 0.1 M of NaOH solution was added to a solution of zinc acetate dihydrate (0.124 mol) containing 10 g of CSS in drops under constant stirring for 30 min. Finally, the mixture was filtered, and the solid product obtained was washed with distilled water and absolute ethanol several times. The nanocomposite (SCZ) obtained was dried at 60 °C for 24 h and calcined at 350 °C for 1 h.

### Instrumentation

Thermogravimetric analysis (TGA) was applied to track the thermal decomposition process of SCZ and SSB using a Mettler Toledo TGA/DSC1 Star System instrument under a nitrogen atmosphere. Spectral for SCZ, SCZ-MTZ, SSB, and SSB-MTZ were acquired using a Fourier transform infrared (FT-IR) spectrophotometer (ThermoFisher Scientific, Waltham, MA, USA). The X-ray diffraction (XRD) pattern of SCZ and SSB was recorded using Bruker D8 Advance powder X-ray diffraction (Bruker, US) using Cu − Kα radiation. The surface morphology of SCZ, SCZ-MTZ, SSB, and SSB-MTZ was obtained using the scanning electron microscope (SEM) (JSM-7500 F, JEOL, Tokyo, Japan). pH points of zero charges of SCZ and SSB were validated via the solid addition method (Mondal and Basu 2019).

### Sorption experiment

The detailed procedures for the batch adsorption of MTZ, the antioxidant analysis, the antimicrobial testing protocol, as well as the kinetics and isotherm models applied, are all provided in the supplementary information.

## Results and discussion

**Fig. 1:**
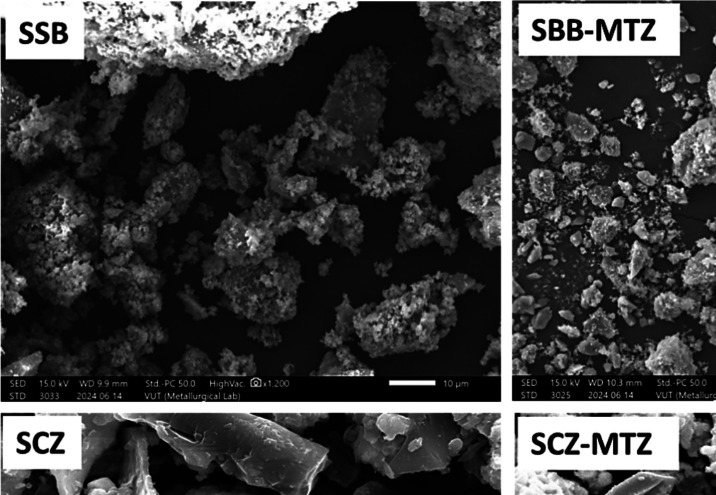
SEM images of SSB, SSB-MTZ, SCZ, and SCZ-MTZ

A SEM micrograph of SSB, SSB-MTZ, SCZ, and SCZ-MTZ was shown in Fig. 1. The pristine snail shell (SSB) revealed the presence of homogenous and irregularly shaped particles, and on application as adsorbent for the adsorption of MTZ, the spent adsorbent (SBB-MTZ) was noticed to sustain similar surface morphology. This indicates that the structural composition of SBB was unaltered after the adsorption step. On the other hand, the surface morphology of SCZ revealed a nonhomogeneous surface-sustaining cavity of varied pore sizes. On application as an adsorbent, the used adsorbent (SCZ-MTZ) exhibited a smooth, irregular spherical characteristic, indicating successful adsorption of MTZ and sustained structural integrity.

**Fig. 2:**
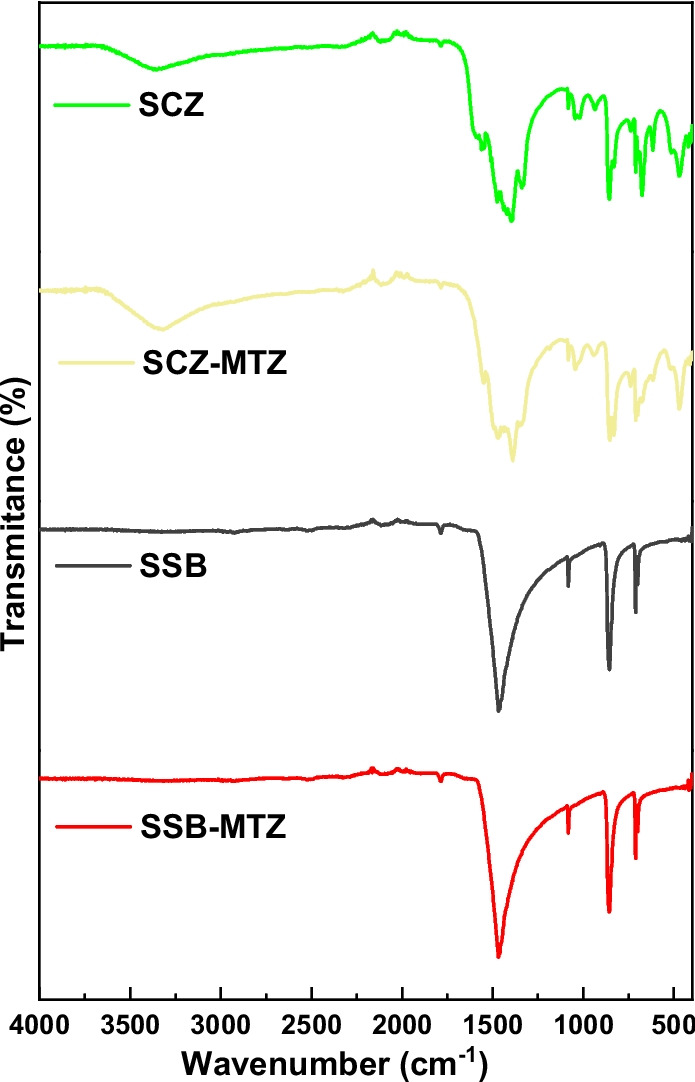
FTIR spectra of SSB, SSB-MTZ, SCZ, SCZ-MTZ

The FTIR spectra of SSB, SSB-MTZ, SCZ, and SCZ-MTZ were used to determine and compare the chemical moieties on the surface of the pristine and spent adsorbents. As displayed in Fig. 2, Broadbands around 3400 cm^−1^ were assigned to hydroxyl groups on the surface of SCZ and SCZ-MTZ. This band arises due to the vibration of the hydroxyl group linked in oxygen functionalities associated with the graphitization of the shell. The peaks observed at 2930 and 1400 cm^−1^ were attributed to the stretching and bending vibration of the C–H bond, respectively. Meanwhile, bands between 1024 and 1083 cm^−1^ were assigned to C–O stretching. Furthermore, peaks at 850 and 450 cm^−1^ may be assigned to C_1_–O–C_4deform_, and Zn–O–Zn stretching, respectively (Kamal et al. 2022; Kaur 2022). Interestingly, the shift in the vibrational bands may be attributed to the uptake of the MTZ onto the adsorbents (see Table 1).

**Table 1:** The observed FTIR Spectral bands (cm^−1^) and assignments

SSB	SSB-MTZ	SCZ	SCZ-AMX	Assignments
-	-	3330	3322	υ(O–H, N–H)
−1457	1468	1412	1468	υ(COO^−^,C = N)
1075	1083	1024	1035	υ_str_(C–O)
851	850	850	841	υ_bend_(C-O)
-	-	457	465	υ_bend_(Si–O-Si, ZnO)

**Fig. 3:**
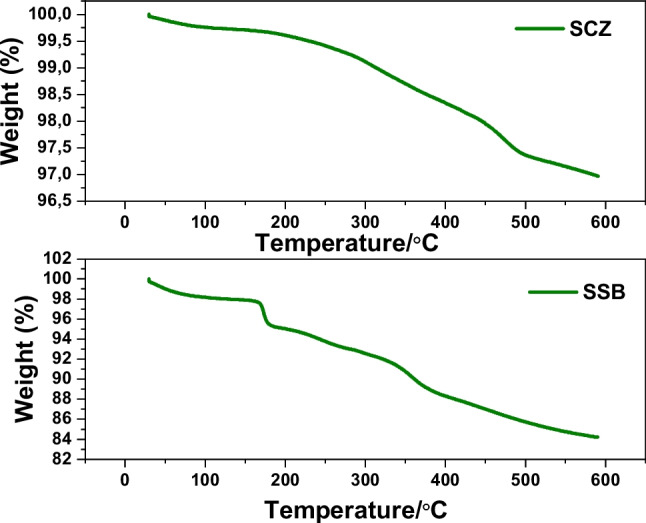
TGA curves of SSB and SCZ

The thermal stabilities of SSB and SCZ were investigated using thermo-gravimetric analysis (TGA), and the outcome is displayed in Fig. 3. With increasing temperatures, SSB and SCZ were noticed to degrade in stages. Two and three stages of degradation were observed for SCZ and SSB, respectively. 0.2% and 2% weight loss were obtained for SCZ and SSB within the 25–150 °C temperature ranges. The observed initial degradation was attributed to the loss of loosely held moisture. The second stage degradation was noticed to lose ~ 3% weight within the temperature range of 150 to 500 °C for SCZ and 150 to 200 °C for BBZ, respectively. The third degradation phase was noticed to lose ~ 4% weight within the temperature range of 200 to 400 °C for SCZ. The weight loss within the second and third thermal treatment phases was ascribed to the thermal decomposition of the hydroxyl and carboxyl constituents of the adsorbents and the evolution of volatile organic and inorganic constituents of SCZ and SSB. Hence, SCZ has demonstrated good adaptability when employed in a thermally elevated environment.

**Fig. 4:**
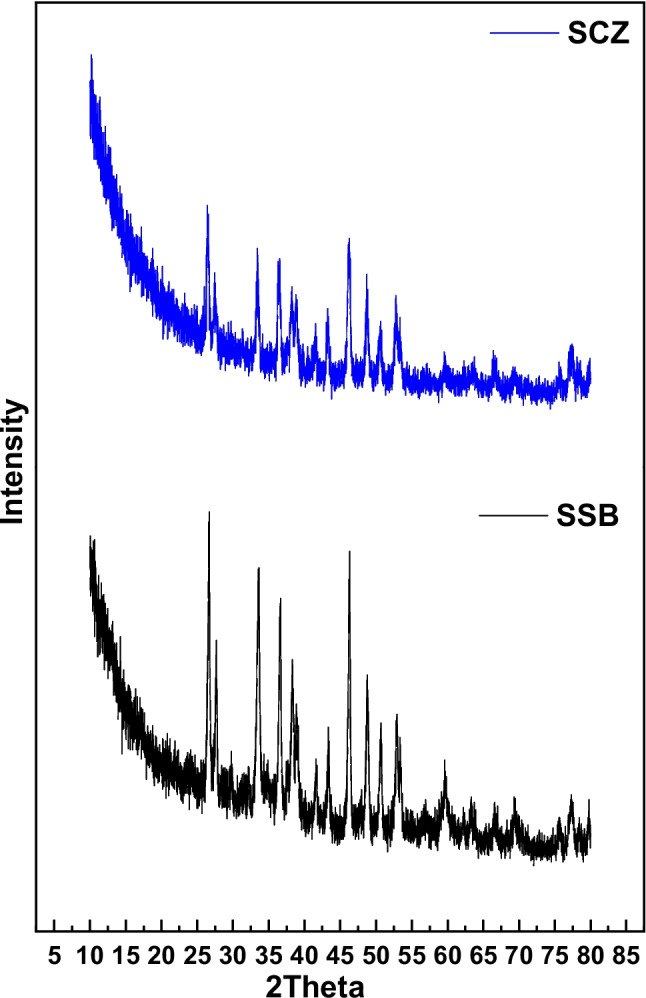
XRD pattern of SSB and SCZ

Snail shells consist mainly of CaCO_3,_ which exists chiefly as calcite or aragonite. Calcite crystallizes with a rhombic system, and aragonite crystallizes with an orthorhombic system (Al Omari et al. 2016). Figure 4 shows the XRD diffractograms of SSB and SCZ. The X-ray diffraction peaks of powdered SSB with relatively large peak intensity show low crystallinity. Many peaks were observed on SSB diffractograms, among which the dominant peaks of 2θ occurred at 26.28°, 33.84°, 36.61°, 38.78°, 41.58°, 43.37°, 46.17°, 48.62°, 50.47°, 52.51°, and 59.40°. The diffractogram acquired for the snail shell was consistent with file JCPDS No. 01–083-0577, suggesting a stable calcite phase (Gunasekaran and Anbalagan 2008; Hossain and Ahmed 2023). On the other hand, the surface fixation of carbon and ZnONPs to the snail shell resulted to reduction of peak intensity of the composite (SCZ) with dominant peaks of 2θ at 26.45°, 33.46°, 36.54°, 38.39°, 41.74°, 43.23°, 46.09°, 48.86°, 50.71°, and 52.78°, suggesting that the structural composition of the snail shell was not destroyed but modified.

### Effect of pH

**Fig. 5:**
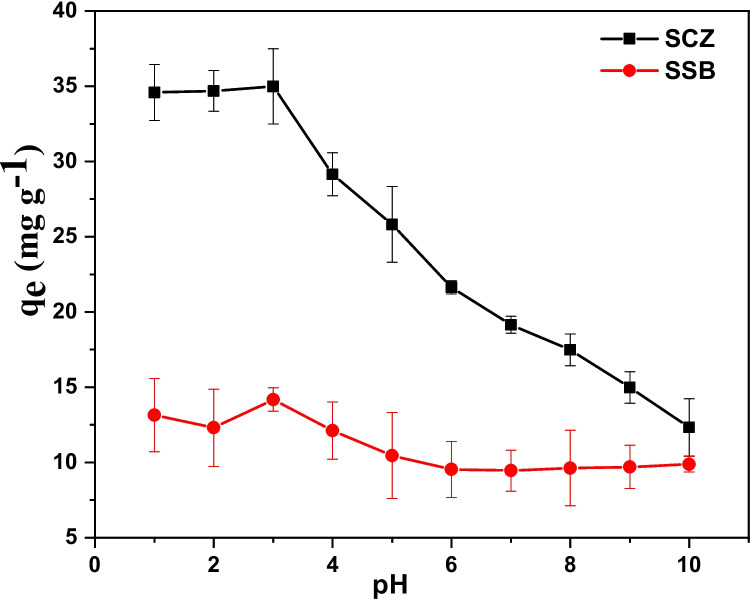
The influence of solution pH on the ability of SSB and SCZ to sequester MTZ

**Fig. 6:**
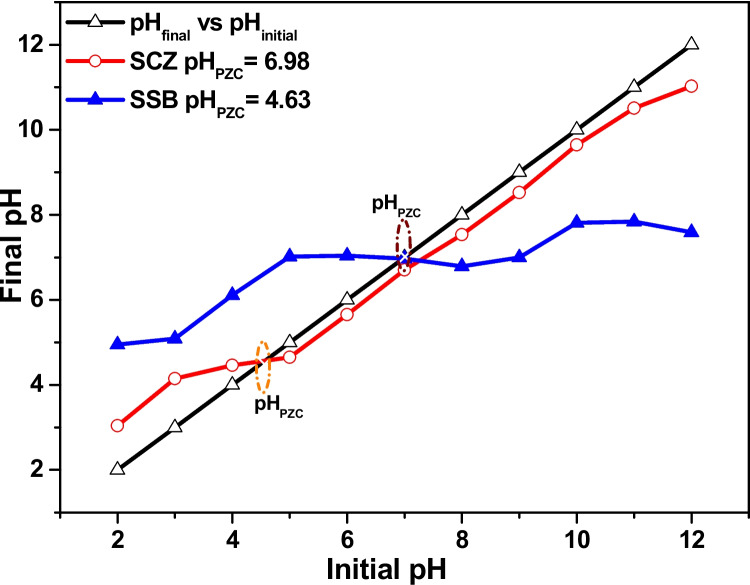
pH_PZC_ plots of SSB and SCZ

SCZ and SSB were used as an adsorbent material to remove metronidazole from the aqueous solution effectively. The implication of solution pH on the capacity of SCZ and SSB to trap metronidazole was investigated by varying the solution pH from pH 2 to 12, while other parameters (0.03 g dosage, 180 min contact time, 50 mg g^−1^ (25 cm^3^) 150 rpm and 298 K solution temperature) were unvaried. Figure 5 demonstrated the capacity of SCZ and SSB to trap metronidazole with variation in solution pH. The graph illustrates the maximum adsorption of metronidazole on SCZ and SSB at pH 3. Above pH 3, the potential of the adsorbent dropped continuously to pH 10. To uncover the mechanism behind the optimum adsorption of metronidazole at pH 3, the pH_PZC_ experiment was performed for SCZ and SSB. From Fig. 6, the pH_PZC_ of SCZ and SSB were extrapolated as 6.98 and 4.63, respectively. Hence, above and below these values, the surface of these materials will be negatively and positively charged, respectively. Interestingly, the pKa of metronidazole is 2.62 (Jonidi Jafari et al. 2024; Mohammadian et al. 2024). Meanwhile, above and below these values, the analyte will be deprotonated and protonated, respectively. Hence, at solution pH 3, metronidazole will be slightly deprotonated (negatively charged) while the surface of SCZ and SSB will be positively charged. It therefore suggests the possibility of electrostatic interaction between the adsorbents and adsorbate interactions in the removal of metronidazole from the aqueous phase.

### The effect of adsorbent dose

**Fig. 7:**
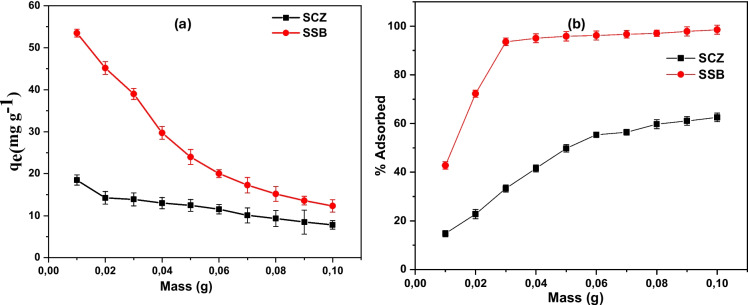
The effect of adsorbent dosage on the sequestration MTZ by SSB and SCZ (**b**) % adsorbed and (**a**) adsorption capacity

The influence of the adsorbent dosage was investigated on the adsorptive removal of metronidazole by varying the mass of the SSB and SCZ adsorbent from 0.01 g to 0.10 g, the result of the investigation is depicted below in Fig. 7. The capacity of SSB and SCZ to sequester metronidazole was noticed to decline from 18.62 to 7.85 mg g^−1^ and 54.61 to 13.25 mg g^−1^, respectively. The reason for this observation is that adsorption sites on SSB and SCZ become unsaturated when the adsorbent dose is increased while maintaining the same metronidazole concentration and volume. On the contrary, the efficiency of SSB and SCZ to sequester metronidazole was noticed to increase with increasing adsorbent dose. A high removal efficiency of metronidazole was obtained for SCZ, with percentages in the range of 48 to 98% being obtained in contrast to 17 to 61% for SSB. The phenomenon is associated with the fact that as the adsorbent dose increases, there is an increase in the surface area and number of active sites available on SSB and SCZ adsorption.

### Adsorption kinetics

**Fig. 8:**
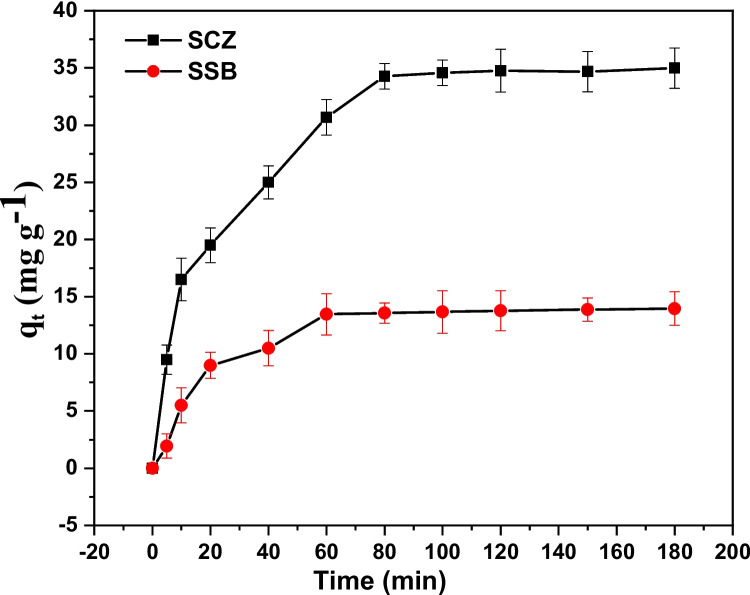
Effect of contact time on the sequestration of MTZ by SSB and SCZ

**Fig. 9:**
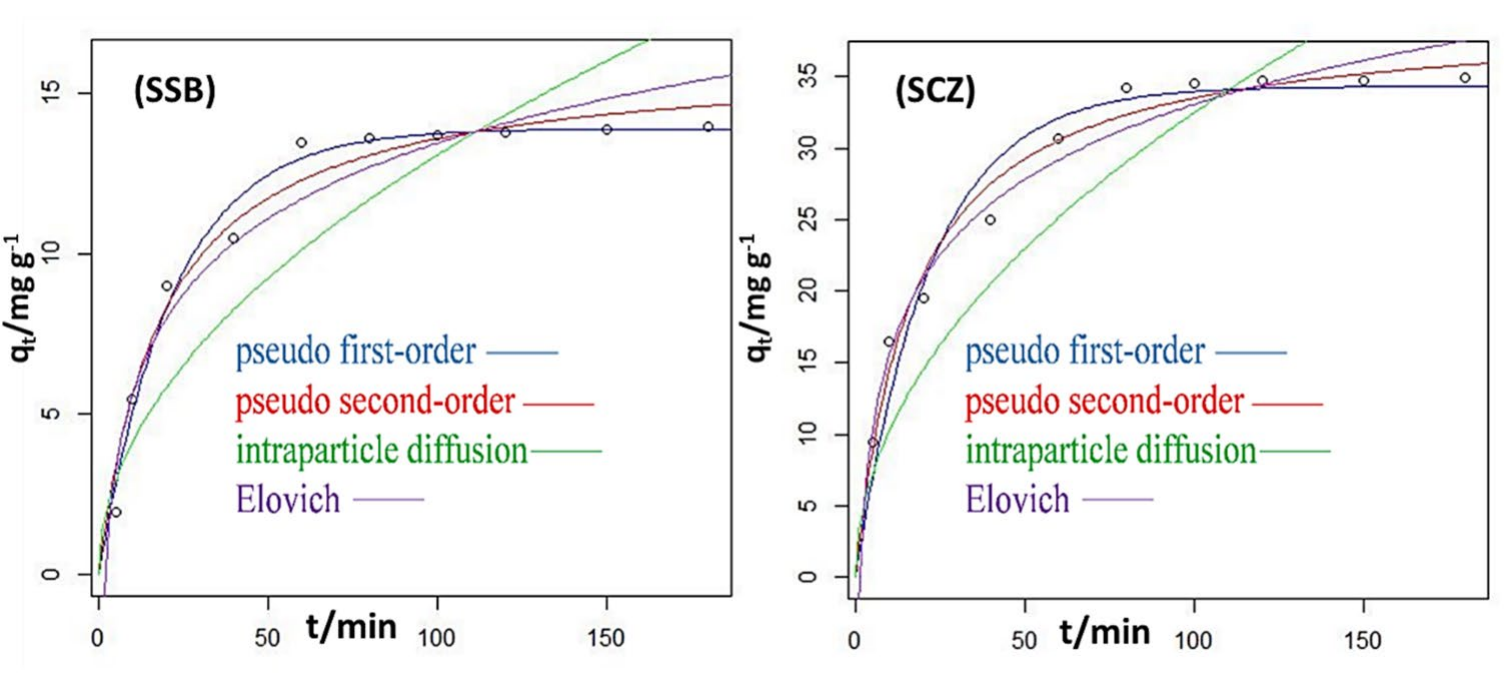
Comparison of the different kinetics models fitted to the experimental data of MTZ uptake onto SSB and SCZ

The implication of contact time on the adsorptive removal of metronidazole was assessed at varying time intervals, between 5 and 180 min, while keeping the initial metronidazole concentration (50 mg dm^−3^), 0.03 g adsorbent dose and a solution pH of 3.0 fixed. The removal efficiency of metronidazole by SSB and SCZ was noticeably increased with time, attaining equilibrium for SCZ and SCZ at 60 min and 80 min, respectively (see Fig. 8). Beyond this stage, the efficiency of SSB and SCZ in trapping metronidazole was noticed to remain steady with little or no further increase. The observation could be attributed to the fact that at the initial stage, more active sites were available for adsorption, therefore facilitating a quicker uptake of metronidazole. There was a marginal uptake of metronidazole when the binding sites on the surface of SSB and SCZ were saturated. Despite the fact that adsorption occurred quickly in both SSB and SCZ, SCZ consistently demonstrated a greater removal potential than SSB. This may be explained by the incorporation of ZnONP into the nanocomposite, which induces degradation cum molecular trapping of metronidazole into its pores. To ensure the attainment of equilibrium, 180 min was employed for further study. Furthermore, pseudo first-order and second-order, Elovich and intra-particle diffusion models were used to unravel the mechanism underlying the uptake of metronidazole by SCZ and SSB (see Table S1 (see supplementary information)). This was achieved by fixing the data acquired from the contact time experiment and integrating it into the aforementioned kinetic model via the nls in the R statistical environment. The models also give insight into the kinetic parameters of the sorption data. Figure 9 reveals the relationship between the uptake potential of SCZ and SSB and contact time. Meanwhile, Table 2 compares the kinetic parameters estimated for the four models. In this study, the least sum of square residual was used to select the model that best describes the adsorption process. The uptake of metronidazole by SCZ (SSR = 19.51) and SSB (SSR = 5.128) was best described by a pseudo-second-order model, indicating that adsorption took place via the bimolecular interactions involving the sharing or exchange of electrons between metronidazole and the adsorbent. This is in good agreement with the effect of pH experiments and previous studies reported by other researchers (Jonidi Jafari et al. 2024; Nazempour et al. 2024).

**Table 2:** The estimated kinetic parameters for the uptake of MTZ onto SSB and SCZ

Model	Parameter	SCZ	SSB
experimental	q_exp_ (mg g^−1^)	35.64	14.35
Pseudo first order	K_1_ (min^−1^)	0.046	0.045
	q_eq_ (mg g^−1^)	34.32	13.88
	SSR	41.10	6.994
	RSE	2.267	0.612
Pseudo second order	K_2_ (g mg^−1^ min^−1^)	0.002	0.003
	q_eq_ (mg g^−1^)	39.19	16.11
	SSR	19.51	5.128
	RSE	1.562	0.801
Intraparticle diffusion	K_id_ (mg g^−1^ min^−0.5^)	3.246	1.305
	l (mg g-^1^)	-	-
	SSR	250.8	50.39
	RSE	5.279	2.366
Elovich	α (mg g^−1^ min^−1^)	−5.916	−2.210
	Β (g mg^−1^)	7.592	3.396
	SSR	26.20	9.935
	RES	1.810	1.114

### Isotherm study

**Fig. 10:**
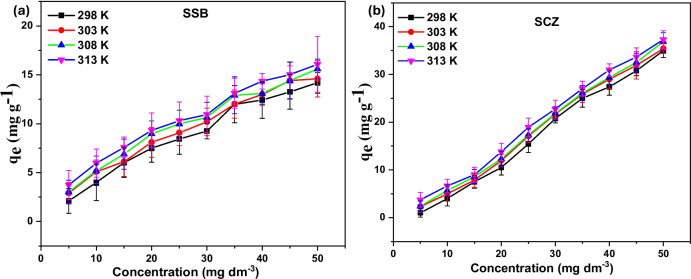
The influence of adsorbate concentration on the uptake of MTZ by (**a**) SSB and (**b**) SCZ

The optimum parameters obtained for dosage (0.03 g), contact time (180 min), and pH (3.0) were used to investigate the effect of varying (5 to 50 mg dm^−3^) initial concentration on the adsorption of metronidazole onto SCZ and SSB. As expected, the uptake potential of SCZ and SSB increased with increasing metronidazole concentrations, which might be associated with the progressive saturation of the adsorbent surface by metronidazole (see Fig. 10). The observed may be attributed to the increased concentration gradient of the adsorbate (Aniagor and Menkiti 2024, Rajabi et al. 2024). At 298 K, the uptake potential of SCZ and SSB increased from 5.24 to 34.71 mg g^−1^ and 7.16 to 13.54 mg g^−1^, respectively, as the initial metronidazole concentration increased from 5 to 50 mg dm^−3^. A similar trend was observed when the experiment was repeated for temperatures 303 K, 308 K, and 313 K, indicating an endothermic adsorption process. Interestingly, SCZ demonstrated promising potential for the removal of MTZ when compared with the capacity of other adsorbents (see Table 3). Isotherm models give insight into the potential of adsorbents or the amount required to eliminate a unit mass of adsorbate under the same experimental conditions(Bbumba et al. 2024; Krishnan et al. 2010). Freundlich and Langmuir’s isotherm models were applied to the acquired equilibrium data. The nonlinear equations of the models are provided in Table S2 (see supplementary information). The model that best describes the equilibrium data was selected based on the least SSR value. Table 4 presents the parameters of the models that best fit the equilibrium data acquired for each SSB and SCZ. Results acquired revealed that the uptake of MTZ onto SSB was best described by the Langmuir isotherm model. This model assumes monolayer adsorption onto homogeneous surfaces with a defined number of identical sites (Alafnan et al. 2021). Hence, MTZ was sorbed onto uniform and equivalent sites on SSB, wherein there exists no interaction between adjacent MTZ molecules. The calculated value of b indicates the adsorptive strength. The study revealed that the adsorptive strength (*b*) for SSB is dependent on solution temperature. This further suggests that strong interactions were formed between MTZ and SSB. The uptake of MTZ onto SCZ was best described by the Freundlich isotherm model. The model assumes a heterogeneous surface which is therefore based on a multi-layer principle of adsorption (Vigdorowitsch et al. 2021). The isotherm analysis revealed the implication of the modification step. Meanwhile, the outcome of this study is consistent with the findings of other authors (Gahrouei et al. 2024; Rajabi et al. 2024).

**Table 3:** Comparing the uptake capacity of the adsorbent used in this study with other adsorbents

Adsorbent type	Absorption capacity (mg g^−1^)	Reference
FeNi_3_/SiO_2_/CuS	135/135	(Nasseh et al. 2019)
Fe-modified sepiolite	5.62	(Ding and Bian 2015)
Canola biomass	42/21	(Sepehr et al. 2017)
LECA	56.31	(Kalhori et al. 2017)
MgO/light-weight expanded clay aggregates	55/84	(Méndez-Díaz et al. 2010)
Kaolin	41.34	(Aleanizy et al. 2015)
SSB	25.97	Present study
SCZ	35.13	Present study

**Table 4:** Adsorption isotherm model parameters for MTZ sequestration by SSB and SCZ

Adsorbent	Isotherm	Parameters		Temperature		
			295 K	303 K	310 K	318 K
SCZ	Freundlich	K_F_	0.37992	0.52822	0.34038	0.39869
		n	0.48267	0.51826	0.50136	0.52730
		SSR	714.000	703.000	720.000	741.000
	Langmuir	qm	33.412	34.1953	34.97496	35.13223
		b	0.21429	0.26248	0.391248	0.63120
		SSR	1106.00	1487.00	3225.00	4246.00
SSB	Freundlich	K_F_	1.50842	1.7793	1.90022	2.07624
		n	1.5426	1.64386	1.6699	1.71879
		SSR	3.7100	3.3799	3.970	3.9139
	Langmuir	qm	24.1823	24.7026	25.09121	25.96737
		b	0.03528	0.04258	0.044487	0.045713
		SSR	3.35412	3.1889	3.7720	3.5288

### Thermodynamics

The thermodynamic characteristics responsible for the uptake of MTZ onto SSB and SCZ were investigated over a varied range of temperatures. The change in Gibbs energy (ΔG°) was calculated from Eq. 4 (Doke and Khan 2013, Milonjić 2007).

1 $$\Delta G^\circ=-RT\;1\mathrm n\;K$$

where *R* is the gas constant (8.314 J K^−1^ mol^−1^), *T* is the temperature in Kelvin, and *K* was calculated from the product of q_m_ and 1000*b.* q_m_ and *b* were parameters estimated from the Langmuir plot (see Table 5). Change in enthalpy (ΔH°) and entropy change (ΔS°) were calculated from the slope and intercept of a linear plot of ln *K* against 1/*T* Eq. 5.

**Table 5:** Thermodynamic factors for absorption of MTZ onto SSB and SCZ

Adsorbents	T/K	ΔG° (kJ mol^−1^)	ΔH (kJ mol^−1^)	ΔS (J K^−1^ mol^−1^)
SCZ	298	−21.9928	59.00	271.16
	303	−22.9312		
	310	−24.3895		
	318	−26.0418		
SSB	298	−17.3465		
	303	−17.5299	1.157	62.25
	310	−17.9714		
	318	−18.7175		

2 $$1\mathrm n\;K=-\frac{\Delta H^\circ}{RT}+\frac{\Delta S^\circ}R$$

Within the range of temperature investigated, negative values of ΔG° were estimated for the adsorption of MTZ, indicating the spontaneous and feasible nature of MTZ adsorption onto SSB and SCZ (see Table 5). It was also noticed that the negative values of ΔG° increased with increasing solution temperature, indicating enhanced MTZ removal as the temperature is increased. Furthermore, positive values of ΔS° suggest an increased degree of disorderliness at the solid–liquid interphase; hence, the uptake of MTZ onto SSB and SCZ was entropy-driven. Interestingly, positive values of ΔH° indicate that the uptake of MTZ onto SSB and SCZ was an endothermic process. On the other hand, adsorption heat between 2.1 and 20.9 kJ mol^−1^ and 80 to 200 kJ mol^−1^ suggest physisorption and chemisorption processes, respectively (Gulab et al. 2024). Hence, the uptake of MTZ onto SSB and SCZ was attributed to physisorption (interaction through weaker van der Waals forces between the MTZ and BBZ) and physicochemical process, respectively.

### Reusability

**Fig. 11:**
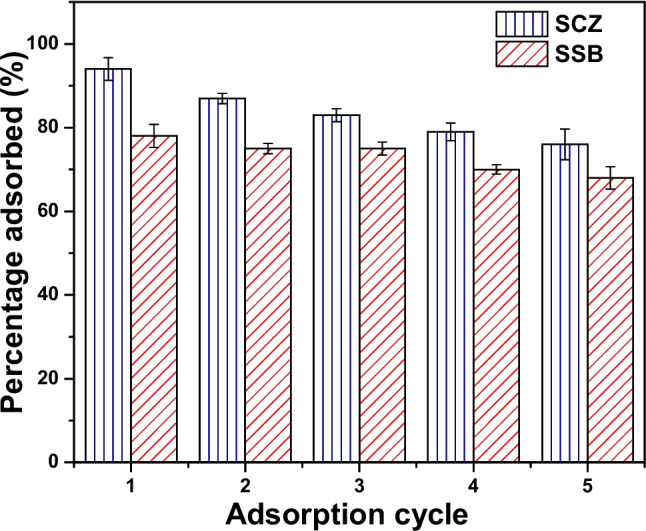
Reusability SSB and SCZ for the uptake of MTZ

Regeneration and reuse of adsorbents are largely dependent on the quality of the eluting agent employed. This step (regeneration) is a significant aspect of the replicability and overall economy of the adsorption process. A preliminary assessment of a suitable eluting agent for the desorption of MTZ was performed to establish the possibility of the reuse of the adsorbent material. Solvents such as deionized water, HCl, methanol, NaOH, and ethanol were investigated for MTZ desorption efficiency. Deionized water, NaOH, HCl, methanol, and ethanol demonstrated 15.42%, 19.26%, 35.36%, 41.26%, and 83.27% desorption efficiency. Studies on adsorption–desorption were conducted utilizing ethanol as the preferred eluting agent. After five regeneration cycles, the uptake efficiency of SSB and SCZ were noticed to decline from 78.72 to 67.21% and from 93.25 to 77.16%, respectively (see Fig. 11). The results presented in Fig. 11 showed the robustness of SCZ after 5 adsorption–desorption cycles, hence, SCZ can be employed for diverse environmental remediation practices.

### Adsorption mechanism

The adsorption of metronidazole onto the ZnONP/carbon-coated snail shell (SCZ) nanocomposite is driven by a synergistic interplay of physical and chemical interactions. Electrostatic attraction serves as a primary mechanism, with the solution pH and the surface charge of SCZ critically influencing the interaction dynamics. π-π electron donor–acceptor interactions are established between the aromatic moieties of the carbon coating and the conjugated system of metronidazole, facilitating strong affinity between the adsorbent and adsorbate. Furthermore, hydrogen bonding between surface functional groupssuch as hydroxyl and carbonyl groups on SCZ and the polar groups in metronidazole, enhances molecular immobilization. In addition, surface complexation through coordination bonds between the ZnO nanoparticles and the electron-rich nitrogen and oxygen atoms of metronidazole significantly strengthens the adsorption capacity. Van der Waals forces provide supplementary physisorption contributions, while the hierarchical porous architecture of the carbon matrix promotes a pore-filling mechanism, further boosting adsorption efficiency. Collectively, these interconnected mechanisms result in the highly effective and stable sequestration of metronidazole by the SCZ nanocomposite.

### Antioxidant potential of SSB and SCZ

**Fig. 12:**
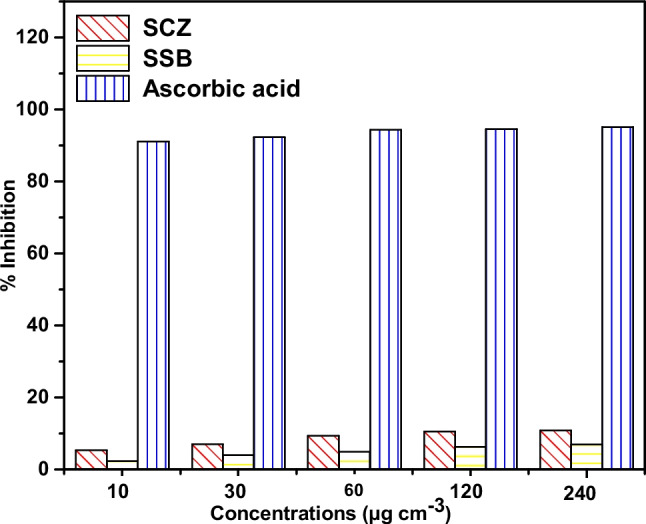
Antioxidant activity of ABZNC and AMB

Every living cell undergoes oxidation by nature, which produces reactive oxygen species (ROS), that can interfere with the cell's regular metabolic processes. Inadequate levels of antioxidants can lead to disastrous outcomes, including lipid peroxidation, DNA and protein damage, and enzyme inactivation (Valgimigli 2023). For SSB and SCZ, the primary DPPH radical scavenging potential was found to be 11.38% and 4.32%, respectively, at the maximal dose of 240 µg cm^−3^ (see Fig. 12). The presence of diverse chemical moieties on the surface of SSB along with the combined synergistic action of ZnONPs, accounts for the robust antioxidant ability of SCZ. The differences between SCZ’s antioxidant activities and those of previous nanoparticle studies could be attributed to a number of factors, including the particle size, the nature of the precursor as well as the experimental procedure employed for synthesis. The findings from the antioxidant study reveal that SCZ exhibits strong antioxidant characteristics.

### Antibacterial study

The inhibitory effect of SSB and SCZ against gram-positive and gram-negative bacteria strains were assessed (see Figs. 1S and 2S). Subsequently, the antimicrobial efficacy of SSB and SCZ was investigated using gram-negative bacteria such as *Escherichia coli* and gram-positive bacteria *Staphylococcus aureus*. SSB and SCZ demonstrate an inhibition zone of 1.0 mm and 2.0 mm against Escherichia coli, respectively. Conversely, SSB and SCZ showed an inhibition zone of R and 6.0 mm against *Staphylococcus aureus*, respectively (see Table 6). The bactericidal activities of SCZ are attributed to its ability to generate toxic ions with the capacity to alter the morphological properties of the membrane resulting in the damage of the cell wall. Hence, the intracellular activity of the cell is compromised, resulting in cell death.

**Table 6:** Antimicrobial activity of SSB and SCZ against *Staphylococcus aureus Escherichia*

Samples	Zone of inhibition (mm)	
	*S. aureus*	*E. coli*
SCZ	6.0	2.0
SSB	R	1.0
Control	20.0	15.0

## Conclusion

This study therefore demonstrates that the incorporation of graphite and ZnONPs into the snail shell can improve the textural characteristics and adsorption potential of SCZ. Spectroscopic techniques such as FTIR, XRD, TGA, and SEM were used to characterize adsorbents. One limitation of this study is the absence of BET surface area analysis, which would provide further insight into the adsorptive properties of the materials. Future work should include surface area measurements to strengthen the understanding of MTZ interaction with SCZ. Adsorption of MTZ from simulated wastewater was successfully performed using SCZ and SSB. The maximum removal of MTZ from simulated wastewater was achieved at an optimum pH of 3, and equilibrium was reached after 180 min. Based on the experimental results obtained, it was observed that the adsorption capacity of SSB and SCZ to remove metronidazole was 25.96 mg g^−1^ and 35.13 mg g^−1^, respectively. The kinetics of adsorption followed the pseudo-second order model indicating a bimolecular rate-determining step. The equilibrium data acquired for SSB and SCZ were better described by the Langmuir and the Freundlich isotherm models, respectively. An increase in the adsorption capacity was achieved as the initial MTZ concentration, contact time, adsorbent dose, and solution temperature were increased. The adsorption process was thermodynamically spontaneous and entropy-driven for the adsorbents. The adsorbed MTZ was efficiently desorbed using ethanol. Thus, SCZ shows potential for reuse and does not create a secondary pollutant problem. The interactions between the MTZ and SSB or SCZ were strong, indicating both physisorption and physicochemical processes. The incorporation of carbon and ZnO nanoparticles significantly improved the metronidazole-binding capacity of SCZ, while also enhancing its antioxidant and antimicrobial properties. Additionally, SCZ exhibited superior reusability and performance compared to SSB. It is interesting to observe the superior adsorption capacity of SCZ over other adsorbents. This study, therefore, demonstrates that ZnONPs/snail shell/carbon nanocomposites can be employed as adsorbents for the elimination of pharmaceuticals from the aquatic environment through adsorption.

## Supplementary Information

Below is the link to the electronic supplementary material.

Supplementary file1 (DOCX 26 KB)

## Data Availability

Not applicable.

## References

[CR1] Abbas H, Abbas AS (2021) Adsorption of flagyl on prepared ash from rice husk. Iraqi J Chem Pet Eng 22:11–17

[CR2] Ahmadfazeli A, Poureshgh Y, Rashtbari Y, Akbari H, Pourali P, Adibzadeh A (2021) Removal of metronidazole antibiotic from aqueous solution by ammonia-modified activated carbon: adsorption isotherm and kinetic study. J Water Sanit Hyg Dev 11:1083–1096

[CR3] Al Omari M, Rashid I, Qinna N, Jaber A, Badwan A (2016) Calcium carbonate. Profiles Drug Subst Excip Relat Methodol 41:31–132 26940168 10.1016/bs.podrm.2015.11.003

[CR4] Alafnan S, Awotunde A, Glatz G, Adjei S, Alrumaih I, Gowida A (2021) Langmuir adsorption isotherm in unconventional resources: applicability and limitations. J Pet Sci Eng 207:109172

[CR5] Aleanizy FS, Alqahtani F, Al Gohary O, El Tahir E, Al Shalabi R (2015) Determination and characterization of metronidazole–kaolin interaction. Saudi Pharm J 23:167–176 25972737 10.1016/j.jsps.2014.06.006 PMC4421081

[CR6] Allohverdi T, Mohanty AK, Roy P, Misra M (2021) A review on current status of biochar uses in agriculture. Molecules 26:5584 34577054 10.3390/molecules26185584 PMC8470807

[CR7] Al-Musawi TJ, Moghaddam NSM, Rahimi SM, Amarzadeh M, Nasseh N (2022) Efficient photocatalytic degradation of metronidazole in wastewater under simulated sunlight using surfactant-and CuS-activated zeolite nanoparticles. J Environ Manage 319:115697 35868191 10.1016/j.jenvman.2022.115697

[CR8] Aniagor CO, Menkiti MC (2024) Analysis of metronidazole adsorption onto cellulose-chitosan composite adsorbent. UNIZIK J Eng Appl Sci 3:1307–1316

[CR9] Asgari E, Sheikhmohammadi A, Yeganeh J (2020) Application of the Fe3O4-chitosan nano-adsorbent for the adsorption of metronidazole from wastewater: optimization, kinetic, thermodynamic and equilibrium studies. Int J Biol Macromol 164:694–706 32702424 10.1016/j.ijbiomac.2020.07.188

[CR10] Bbumba S, Karume I, Nsamba HK, Kigozi M, Kato M (2024) An insight into isotherm models in physical characterization of adsorption studies. European Journal of Applied Sciences 2:12

[CR11] Caneschi A, Bardhi A, Barbarossa A, Zaghini A (2023) The use of antibiotics and antimicrobial resistance in veterinary medicine, a complex phenomenon: a narrative review. Antibiotics 12:487 36978354 10.3390/antibiotics12030487 PMC10044628

[CR12] Cheng N, Wang B, Wu P, Lee X, Xing Y, Chen M, Gao B (2021) Adsorption of emerging contaminants from water and wastewater by modified biochar: a review. Environ Pollut 273:116448 33486256 10.1016/j.envpol.2021.116448

[CR13] Chua KYL (2017) Metronidazole, Kucers' the use of antibiotics. CRC Press, pp 1807–1849

[CR14] de Jesus GV, Almeida CM, Rodrigues A, Ferreira E, Benoliel MJ, Cardoso VV (2015) Occurrence of pharmaceuticals in a water supply system and related human health risk assessment. Water Res 72:199–208 25453834 10.1016/j.watres.2014.10.027

[CR15] Ding H, Bian G (2015) Adsorption of metronidazole in aqueous solution by Fe-modified sepiolite. Desalin Water Treat 55:1620–1628

[CR16] Doke KM, Khan EM (2013) Adsorption thermodynamics to clean up wastewater; critical review. Rev Environ Sci Bio/technol 12:25–44

[CR17] Egea-Corbacho A, Ruiz SG, Alonso JMQ (2019) Removal of emerging contaminants from wastewater using nanofiltration for its subsequent reuse: full–scale pilot plant. J Clean Prod 214:514–523

[CR18] El Bouraie MM, Ibrahim SS (2021) Comparative study between metronidazole residues disposal by using adsorption and photodegradation processes onto MgO nanoparticles. J Inorg Organomet Polym Mater 31:344–364 10.2147/IJN.S265739 PMC7533914 33061371

[CR19] El-Sayed G, Dessouki H, Jahin H, Ibrahiem S (2014) Photocatalytic degradation of metronidazole in aqueous solutions by copper oxide nanoparticles. J Basic Environ Sci 1:102–110

[CR20] Gahrouei AE, Vakili S, Zandifar A, Pourebrahimi S (2024) From wastewater to clean water: recent advances on the removal of metronidazole, ciprofloxacin, and sulfamethoxazole antibiotics from water through adsorption and advanced oxidation processes (AOPs). Environ Res. 10.1016/j.envres.2024.119029 10.1016/j.envres.2024.119029 38685299

[CR21] Gil A, Taoufik N, García A, Korili SA (2019) Comparative removal of emerging contaminants from aqueous solution by adsorption on an activated carbon. Environ Technol. 10.1080/09593330.2018.1464066 10.1080/09593330.2018.1464066 29634434

[CR22] Gulab H, Malik S, Jan L, Idrees M, Gohar O (2024) Adsorptive removal of Famotidine drug from aqueous medium by *Ocimum**basilicum*. Chem Select 9:e202400255

[CR23] Gunasekaran S, Anbalagan G (2008) Spectroscopic study of phase transitions in natural calcite mineral. Spectrochim Acta A Mol Biomol Spectrosc 69:1246–1251 17913574 10.1016/j.saa.2007.06.036

[CR24] Gupta M, Savla N, Pandit C, Pandit S, Gupta PK, Pant M, Khilari S, Kumar Y, Agarwal D, Nair RR (2022) Use of biomass-derived biochar in wastewater treatment and power production: a promising solution for a sustainable environment. Sci Total Environ 825:153892 35181360 10.1016/j.scitotenv.2022.153892

[CR25] Hossain MS, Ahmed S (2023) Crystallographic characterization of naturally occurring aragonite and calcite phase: rietveld refinement. J Saudi Chem Soc 27:101649

[CR26] Jakimska A, Kot-Wasik A, Namieśnik J (2014) The current state-of-the-art in the determination of pharmaceutical residues in environmental matrices using hyphenated techniques. Crit Rev Anal Chem 44:277–298 25391566 10.1080/10408347.2013.835244

[CR27] Jonidi Jafari A, Jafari Mansoorian H, Askarpour H, Salari M, Eslami F, Faraji M, Shomoossi F, Abdipour H, Jaberi Ansari F (2024) Analyzing and optimizing the adsorption of metronidazole antibiotic on nano-scale pumice mine waste based RSM-CCD technique in water. Int J Environ Sci Technol. 10.1007/s13762-024-06102-9

[CR28] Kalhori EM, Al-Musawi TJ, Ghahramani E, Kazemian H, Zarrabi M (2017) Enhancement of the adsorption capacity of the light-weight expanded clay aggregate surface for the metronidazole antibiotic by coating with MgO nanoparticles: studies on the kinetic, isotherm, and effects of environmental parameters. Chemosphere 175:8–20 28211338 10.1016/j.chemosphere.2017.02.043

[CR29] Kalhorizadeh T, Dahrazma B, Zarghami R, Mirzababaei S, Kirillov AM, Abazari R (2022) Quick removal of metronidazole from aqueous solutions using metal–organic frameworks. New J Chem 46:9440–9450

[CR30] Kamal A, Haroon U, Manghwar H, Alamer KH, Alsudays IM, Althobaiti AT, Iqbal A, Akbar M, Farhana AM (2022) Biological applications of ball-milled synthesized biochar-zinc oxide nanocomposite using Zea mays L. Molecules 27:5333 36014570 10.3390/molecules27165333 PMC9412314

[CR31] Kamali M, Appels L, Kwon EE, Aminabhavi TM, Dewil R (2021) Biochar in water and wastewater treatment-a sustainability assessment. Chem Eng J 420:129946

[CR32] Kariim I, Abdulkareem A, Abubakre O (2020) Development and characterization of MWCNTs from activated carbon as adsorbent for metronidazole and levofloxacin sorption from pharmaceutical wastewater: kinetics, isotherms and thermodynamic studies. Sci Afr 7:e00242

[CR33] Kaur H (2022) Synergistic effect of biochar impregnated with ZnO nano-flowers for effective removal of organic pollutants from wastewater. Appl Surf Sci Adv 12:100339

[CR34] Krishnan KA, Sreejalekshmi K, Varghese S (2010) Adsorptive retention of citric acid onto activated carbon prepared from Havea braziliansis sawdust: kinetic and isotherm overview. Desalination 257:46–52

[CR35] Krishnan KA, Suresh SS, Arya S, Sreejalekshmi KG (2015) Adsorptive removal of 2, 4-dinitrophenol using active carbon: kinetic and equilibrium modeling at solid–liquid interface. Desalin Water Treat 54:1850–1861

[CR36] Krishnan KA, Sreejalekshmi KG, Dev VV, Antony S, Mahadevan H (2017) Removal of Cu (II) from aqueous phase using tailor made sulfur-impregnated activated carbon inspired by Claus process. Desalin Water Treat 80:214–222

[CR37] Manjunath S, Kumar SM, Ngo HH, Guo W (2017) Metronidazole removal in powder-activated carbon and concrete-containing graphene adsorption systems: estimation of kinetic, equilibrium and thermodynamic parameters and optimization of adsorption by a central composite design. J Environ Sci Health A 52:1269–1283 10.1080/10934529.2017.1357406 28920773

[CR38] Matamoros V, Salvadó V (2013) Evaluation of a coagulation/flocculation-lamellar clarifier and filtration-UV-chlorination reactor for removing emerging contaminants at full-scale wastewater treatment plants in Spain. J Environ Manage 117:96–102 23353882 10.1016/j.jenvman.2012.12.021

[CR39] Méndez-Díaz J, Prados-Joya G, Rivera-Utrilla J, Leyva-Ramos R, Sánchez-Polo M, Ferro-García M, Medellín-Castillo N (2010) Kinetic study of the adsorption of nitroimidazole antibiotics on activated carbons in aqueous phase. J Colloid Interface Sci 345:481–490 20193953 10.1016/j.jcis.2010.01.089

[CR40] Milonjić SK (2007) A consideration of the correct calculation of thermodynamic parameters of adsorption. J Serb Chem Soc 72:1363–1367

[CR41] Mohammadian N, Firozjaee TT, Abdi J, Moghadasi M, Mirzaei M (2024) PW12/Fe3O4/biochar nanocomposite as an efficient adsorbent for metronidazole removal from aqueous solution: synthesis and optimization. Surf Interface Anal 52:104946

[CR42] Mondal NK, Basu S (2019) Potentiality of waste human hair towards removal of chromium (VI) from solution: kinetic and equilibrium studies. Appl Water Sci 9:1–8

[CR43] Moreira JB, Santos TD, Zaparoli M, de Almeida ACA, Costa JAV, de Morais MG (2022) An overview of nanofiltration and nanoadsorption technologies to emerging pollutants treatment. Appl Sci 12:8352

[CR44] Morin-Crini N, Lichtfouse E, Liu G, Balaram V, Ribeiro ARL, Lu Z, Stock F, Carmona E, Teixeira MR, Picos-Corrales LA (2022) Worldwide cases of water pollution by emerging contaminants: a review. Environ Chem Lett 20:2311–2338

[CR45] Muneer A, AL-Shemary RQ, Kareem ET (2018) Study on the use of snail shell as adsorbent for the removal of Azure A dye from aqueous solution. Int J Pharm Res 45:123–129

[CR46] Nasseh N, Barikbin B, Taghavi L, Nasseri MA (2019) Adsorption of metronidazole antibiotic using a new magnetic nanocomposite from simulated wastewater (isotherm, kinetic and thermodynamic studies). Compos Part B Eng 159:146–156

[CR47] Nazempour F, Mowla D, Qaretapeh MZ, Dashtian K (2024) Ex-situ magnetic CN-rich activated carbon derived from *Prunus scoparia* shells for the simultaneous adsorption of metronidazole and acetaminophen: kinetic, isotherm, thermodynamic, and multivariate studies. J Environ Chem Eng 12:113762

[CR48] Neghi N, Kumar M, Burkhalov D (2019) Synthesis and application of stable, reusable TiO2 polymeric composites for photocatalytic removal of metronidazole: removal kinetics and density functional analysis. Chem Eng J 359:963–975

[CR49] Patel OP, Jesumoroti OJ, Legoabe LJ, Beteck RM (2021) Metronidazole-conjugates: a comprehensive review of recent developments towards synthesis and medicinal perspective. Eur J Med Chem 210:112994 33234343 10.1016/j.ejmech.2020.112994

[CR50] Pourhakkak P, Taghizadeh M, Taghizadeh A, Ghaedi M (2021) Adsorbent, interface science and technology. Elsevier, pp 71–210

[CR51] Primo JdO, Bittencourt C, Acosta S, Sierra-Castillo A, Colomer J-F, Jaerger S, Teixeira VC, Anaissi FJ (2020) Synthesis of zinc oxide nanoparticles by ecofriendly routes: adsorbent for copper removal from wastewater. Front Chem 8:571790 33330360 10.3389/fchem.2020.571790 PMC7732540

[CR52] Rajabi S, Derakhshan Z, Hashemi M, Feilizadeh M, Heidari Kochaki S, Hashemi H, Salehi M, Zare A, Shourabi NS, Moradalizadeh S (2024) Metronidazole adsorption by bio-synthesized silver-zinc ferrite nanoadsorbent in presence of chitosan from aqueous media: response surface methodology. Appl Water Sci 14:92

[CR53] Rathi BS, Kumar PS (2021) Application of adsorption process for effective removal of emerging contaminants from water and wastewater. Environ Pollut 280:116995 33789220 10.1016/j.envpol.2021.116995

[CR54] Saidulu D, Gupta B, Gupta AK, Ghosal PS (2021) A review on occurrences, eco-toxic effects, and remediation of emerging contaminants from wastewater: special emphasis on biological treatment based hybrid systems. J Environ Chem Eng 9:105282

[CR55] Sarfi S, Azaryan E, Hanafi-Bojd MY, Emadian Razavi F, Naseri M (2024) Green synthesis of nanohydroxyapatite with *Elaeagnus**angustifolia* L. extract as a metronidazole nanocarrier for in vitro pulpitis model treatment. Sci Rep 14:14702 38926433 10.1038/s41598-024-65582-4 PMC11208562

[CR56] Schmidt H-P, Wilson K (2012) 55 uses of biochar. Ithaka Journal 1:286–289

[CR57] Sepehr MN, Al-Musawi TJ, Ghahramani E, Kazemian H, Zarrabi M (2017) Adsorption performance of magnesium/aluminum layered double hydroxide nanoparticles for metronidazole from aqueous solution. Arab J Chem 10:611–623

[CR58] Siddiqi KS, ur Rahman A, Tajuddin N, Husen A (2018) Properties of zinc oxide nanoparticles and their activity against microbes. Nanoscale Res Lett 13:1–13 29740719 10.1186/s11671-018-2532-3 PMC5940970

[CR59] Szentmihályi K, Klébert S, May Z, Bódis E, Mohai M, Trif L, Feczkó T, Károly Z (2022) Immobilization of metronidazole on mesoporous silica materials. Pharmaceutics 14:2332 36365150 10.3390/pharmaceutics14112332 PMC9699156

[CR60] Thiengo SC, Maldonado A, Mota EM, Torres EJL, Caldeira R, Carvalho OdS, Oliveira APMd, Simões RO, Fernandez M, Lanfredi RM (2010) The giant African snail *Achatina fulica* as natural intermediate host of *Angiostrongylus cantonensis* in Pernambuco, northeast Brazil. Acta Trop 115:194–199 20083081 10.1016/j.actatropica.2010.01.005

[CR61] Ukanwa KS, Patchigolla K, Sakrabani R, Anthony E, Mandavgane S (2019) A review of chemicals to produce activated carbon from agricultural waste biomass. Sustainability 11:6204

[CR62] Valgimigli L (2023) Lipid peroxidation and antioxidant protection. Biomolecules 13:1291 37759691 10.3390/biom13091291 PMC10526874

[CR63] Vigdorowitsch M, Pchelintsev A, Tsygankova L, Tanygina E (2021) Freundlich isotherm: an adsorption model complete framework. Appl Sci 11:8078

[CR64] Wang J, Wang S (2019) Preparation, modification and environmental application of biochar: a review. J Clean Prod 227:1002–1022

[CR65] Wu H-Y, Chen SS, Liao W, Wang W, Jang M-F, Chen W-H, Ahamad T, Alshehri SM, Hou C-H, Lin K-S (2020) Assessment of agricultural waste-derived activated carbon in multiple applications. Environ Res 191:110176 32950515 10.1016/j.envres.2020.110176

[CR66] Yu H-W, Park M, Wu S, Lopez IJ, Ji W, Scheideler J, Snyder SA (2019) Strategies for selecting indicator compounds to assess attenuation of emerging contaminants during UV advanced oxidation processes. Water Res 166:115030 31494487 10.1016/j.watres.2019.115030

