# Supplementary material for: Highly effective and reusable nanometal/carbon-coated snail shell for the sequestration of metronidazole: decontamination and disinfection
Source: Environ Sci Pollut Res Int. 2025 Aug 29;32(35):21006–19. doi: 10.1007/s11356-025-36801-w (PMC12443943; doi:10.1007/s11356-025-36801-w)
Supplement: Supplementary file 1 — Supplementary file1 (DOCX 26 KB) [file 11356_2025_36801_MOESM1_ESM.docx]

**Highly effective and reusable nanometal/carbon-coated snail shell for the sequestration of metronidazole: Decontamination and disinfection**

**^*^****James Friday Amaku^1^ · Fanyana M Mtunzi Mtunzi^1^**

^1^Vaal University of Technology, Vanderbijlpark 1911, Gauteng, South Africa

*Corresponding author: fridaya@vut.ac.za

**Antibacterial activity**

The antibacterial properties of SCZ and SSB were evaluated using the Agar-well diffusion method. In the study, ciprofloxacin was used as a positive control and the water treatment agents (SCZ and SSB ) were assessed against gram-negative (*Escherichia coli*) and gram-positive (*Staphylococcus aureus*) harmful bacteria. Muller-Hinton Agar Medium was used to inoculate microbial suspensions in petri plates. Wells around 10 mm in diameter that were bored with a well cutter were filled with about 250 μg of SCZ or SSB samples to be in contact with the bacterial cultures. For 24 h at 37 °C the plates were incubated, and the zone of inhibition that was developed around the well was used to measure the antibacterial characteristics of SCZ and SSB ([Qamar et al. 2017](#_ENREF_55)).

**Antioxidant assay**

Using the DPPH assay, the antioxidant properties of SCZ and SSB were evaluated. In summary, 0.5 cm^3^ of DPPH (0.3 mM) solution was added to SCZ or SSB at various concentrations (10, 30, 60, 120, and 240 g cm^-1^). DPPH is used as a radical source, while the wastewater treatment agent (SCZ and SSB ) serves as a radical scavenger. The mixture of the radical source and the radical scavengers was incubated for 30 minutes at 25°C in a dark container. By utilizing the change in the percentage of absorption wavelength at 517 nm, the concentration of the radical was calculated and the level of inhibition (% I) of the DPPH was determined using **Eqn S1** ([Begum et al. 2022](#_ENREF_12)).

$I\%=\frac{\left( {Asorbance}_{control}-{Asorbance}_{Sample} \right)}{{Asorbance}_{control}}\times100$ (S1)

**Batch adsorption experiments**

The adsorption of metronidazole (MTZ) was achieved in a stoppered amber glass bottle which contained 25 cm3 of metronidazole solution at different concentrations ranging from 5 to

50 mg dm^-3^ and 0.03 g mg of SCZ or SSB with appropriate pH adjusted by using 0.1 mol dm^-3^ NaOH or 0.1 mol dm^-3^ HCl solutions. The kinetics study of the removal process was investigated by varying the contact time from 5-180 min with every other parameter kept constant. The implementation of adsorbent dose (0.01-0.1 g), initial MTZ concentrations (10-100 mg dm^-3^), and solution temperature (298, 303, 308, 313 K) were also assessed. In the overall adsorption process, SCZ or SSB were contacted with MTZ and agitated for 180 min in a thermostated shaking water bath (agitation speed of 120 rpm) at a fixed temperature of 25 °C, thereafter the mixtures were filtered and the final concentration was of MTZ were determined by a UV–visible spectrophotometry (Shimadzu UV-3600) (λ = 273 nm).

The amount of MTZ removed per gram of SCZ or SSB and the removal efficiency (% adsorbed) of MTZ were estimated using Eqns (S2) and (S3) respectively:

$q_{eq}=\left( \frac{C_{i}-C_{eq}}{m} \right)V$ (S2)

$\% adsorbed=\left( \frac{C_{i}-C_{eq}}{C_{i}} \right)\times100$ (S3)

where C_i_ is the initial concentration (mg dm^-3^) of MTZ, C_eq_ is the equilibrium concentration (mg dm^-3^), m is the mass (g) of SCZ or SSB, and V is the volume of MTZ solution (dm^3^). The kinetics data were used to unveil the mechanism responsible for the uptake of MTZ onto SCZ or SSB via data fitting into pseudo-first-order ([Aksu and Karabayır 2008](#_ENREF_3)), pseudo-second-order ([Sevim et al. 2011](#_ENREF_62)), intraparticle diffusion ([Ofomaja, Naidoo, and Modise 2009](#_ENREF_50)), and Elovich ([Omorogie et al. 2016](#_ENREF_51))kinetics models (see **Table S1**). A similar analysis was performed to determine the isotherm and thermodynamic characteristics of the overall process using Freundlich and Langmuir isotherm models (see **Table S2**)

**Table S1** Kinetics models used to assess the uptake of MTZ onto SCZ or SSB

| Kinetic models | Equations | Parameters | References |
| --- | --- | --- | --- |
| Pseudo-first order | $\frac{{dq}_{t}}{d_{t}}=k_{1}\left( q_{e}-q_{t} \right)$ | $q_{e}{,k}_{1}$ | ([Aksu and Karabayır 2008](#_ENREF_3)) |
| Pseudo-second order | $\frac{{dq}_{t}}{d_{t}}=k_{2}\left( q_{e}-q_{t} \right)^{2}$ | $k_{2},q_{e}$ | ([Sevim et al. 2011](#_ENREF_62)) |
| Weber-Morris intraparticle diffusion | $\frac{{dq}_{t}}{d_{t^{-0.5}}}=k_{id}$ | $k_{id,} l$ | ([Ofomaja, Naidoo, and Modise 2009](#_ENREF_50)) |
| Elovich | $\frac{{dq}_{t}}{d_{t}}=\alpha exp\left( -\beta q_{t} \right)$ | $\alpha, \beta$ | ([Omorogie et al. 2016](#_ENREF_51)) |
| α, adsorption rate constant (mg g^-1^ min^-1^); k_1_, pseudo-first order rate constant (min^-1^);. k_id_, intraparticle diffusion rate constant (mg g^-1^ min^0.5^); q_e_, quantity of adsorbate adsorbed at equilibrium (mg g^-1^); l, is a constant related to the boundary layer thickness (mg g^-1^); k_2_, pseudo-second-order rate constant (g mg^-1^ min^-1^) q_t_, quantity of adsorbate adsorbed at time t (mg g^-1^); β, desorption rate constant (g mg^-1^). | | | |

**Table S2** Isotherm equations and parameters used to describe the uptake of MTZ onto SCZ or SSB

| Isotherm model | Equation | Parameters | References |
| --- | --- | --- | --- |
| Langmuir | $q_{e}=\frac{q_{max}{bC}_{e}}{1+bC_{e}}$ | $q_{max},$b | ([Langmuir 1918](#_ENREF_37)) |
| Freundlich | $q_{e}=K_{F}C_{e}^{\frac{1}{n}}$ | $k_{F}, n$ | ([Freundlich 1906](#_ENREF_22)) |

q_eq_, adsorption capacity (mg g^-1^) of SCZ or SSB; C_eq_, equilibrium concentration of RhB in solution (mg dm^-3^); q_max_, maximum monolayer potential (mg g^-1^) of SCZ and SSB; b, Langmuir isotherm constant (dm^3^ mg^-1^); K_F_, Freundlich isotherm constant (mg g^-1^) (dm^-3^ mg^-1^)^n^; n, adsorption intensity.

**Data analysis**

The experimental data obtained from contact time and effect of concentration and temperature experiments were fitted into the kinetics and isotherm models via the NLS nonlinear regression routine in the R statistical computing environment ([Team 2014](#_ENREF_65)). The residuals of the models employed for this study were calculated and used to validate the adequacy of the models.
